# Urinary Metabolomic Profiling after Administration of Corydalis Tuber and Pharbitis Seed Extract in Healthy Korean Volunteers

**DOI:** 10.3390/pharmaceutics13040522

**Published:** 2021-04-09

**Authors:** Hyeon-Cheol Jeong, Jung Eun Park, Yohan Seo, Min-Gul Kim, Kwang-Hee Shin

**Affiliations:** 1 Research Institute of Pharmaceutical Sciences, College of Pharmacy, Kyungpook National University, Daegu 41566, Korea; houkiboshi01@knu.ac.kr (H.-C.J.); seoyo123@knu.ac.kr (Y.S.); 2 New Drug Development Center, Daegu 41061, Korea; parkje6605@dgmif.re.kr; 3 Center for Clinical Pharmacology and Biomedical Research Institute, Jeonbuk National University Hospital, Jeonju 54907, Korea; 4 Department of Pharmacology, School of Medicine, Jeonbuk National University, Jeonju 54907, Korea; 5 Research Institute of Clinical Medicine of Jeonbuk National University, Jeonju 54907, Korea

**Keywords:** DA-9701, prokinetic agents, natural product extracts, metabolomics, HRMS

## Abstract

Pharmacometabolomics is a useful tool to identify biomarkers that can assess and predict response after drug administration. The primary purpose of pharmacometabolomics is to better understand the mechanisms and pathways of a drug by searching endogenous metabolites that have significantly changed after drug administration. DA-9701, a prokinetic agent, consists of Pharbitis seed and Corydalis tube extract and it is known to improve the gastrointestinal motility. Although the overall mechanism of action of DA-9701 remains unclear, its active ingredients, corydaline and chlorogenic acid, act as a 5-HT3 and D2 receptor antagonist and 5-HT4 receptor agonist. To determine the significant metabolites after the administration of DA-9701, a qualitative analysis was carried out using ultra-high performance liquid chromatography coupled with orbitrap mass spectrometer followed by a multivariate analysis. Seven candidates were selected and a statistical analysis of fold change was performed over time. Our study concluded that all the seven selected metabolites were commonly involved in lipid metabolism and purine metabolism.

## 1. Introduction

Functional dyspepsia (FD) is defined as “a discomfort or pain in the upper part of the abdomen” [1]. It is known that the inflammation of the gastrointestinal tract, delayed gastric emptying, and increase of epithelial permeability are caused by various biological and environmental factors, such as changes in the normal intestinal flora, and genetic and psychosocial factors [2]. A study on the Greek population confirmed that CD14, GNB3, MIF, and TRPV1 gene polymorphisms were related to a higher susceptibility of epigastric pain syndrome [3]. As a result of a survey conducted on 3399 Koreans without organic diseases, ~20% (*n* = 694) complained of dyspeptic symptoms such as epigastric pain or postprandial discomfort. It is also believed that FD affects many people around the globe [4].

Proton pump inhibitors, H_2_-receptor antagonists, and prokinetic agents are used to treat FD [5]. Cisapride, a gastrointestinal prokinetic agent, was developed for the treatment of FD and has been widely used; however, severe arrhythmias, such as Torsades de pointes, have been reported as one of its side effects [6]. Although new prokinetic agents have been developed, they show side effects such as cardiac arrhythmia and some had failed to show enough efficacy [7]. Therefore, there is a growing need to develop safer and more effective prokinetic agents.

Corydalis tuber (the root of *Corydalis yanhusuo*) and Pharbitidis seed (the seed of *Pharbitis nil* L. Choisy) have been used in Chinese medicine for the treatment of gastric ulcer [8]. DA-9701 is a prokinetic agent that is formulated with Corydalis tuber and Pharbitis seed. Corydaline and chlorogenic acid, the active ingredients of DA-9701, are known to act as 5-HT_3_ and D_2_ receptor antagonists and 5-HT_4_ receptor agonists [9]. Although exact mechanism was unclear, these mechanisms are considered to improve symptoms of functional dyspepsia and abdominal pain. In previous research, DA-9701 showed an improvement of the delayed gastric emptying and an increasing of the basal gastric volume in mouse or rat with functional dyspepsia [10,11,12,13].

Endogenous metabolites are the final products of the regulation at the cellular level and are known to be influenced by environmental and genetic factors [14]. Metabolomics can aid the identification of biomarkers to diagnose diseases or to evaluate prognosis by analyzing and quantifying changes of metabolites, according to a specific disease state or environmental difference [14]. Pharmacometabolomics is an approach to explore biomarkers that can assess drug response by analyzing and comparing endogenous metabolites change before and after drug administration. Several studies rely on information that metabolomics provides when a single chemical drug is administered [15]; however, there is a lack of research and focus on natural product extracts. The study aimed to investigate and analyze the changes of endogenous metabolites after administration of DA-9701.

## 2. Materials and Methods

### 2.1. Reagents

Methanol and deionized water of LC-MS grade was purchased from Sigma-Aldrich (St. Louis, MO, USA). Formic acid (extra pure grade) was purchased from Duksan (Seoul, Korea). Analytical grade reference compounds, l-acetylcarnitine, azelaic acid, ophthalmic acid, uric acid, suberic acid, ε-(γ-glutamyl)-lysine, and pimelic acid, were purchased from Sigma-Aldrich and Toronto Research Chemicals (Toronto, ON, Canada). Deionized water for sample preparation was obtained using Milli-Q (Merck, Darmstadt, Germany).

### 2.2. Clinical Study and Sample Collection

The clinical study was conducted upon approval of the Institutional Review Board of Chonbuk University Hospital (IRB No. CUH 2016-01-021). A total of 16 subjects participated voluntarily in this clinical study. All volunteers were divided into three groups, and demographical characteristics were presented at Table 1. The first group took a single dose 90 mg of DA-9701 once after fasting (*n* = 4) and the second group was divided into two subgroups of six patients (*n* = 12). The first subgroup received a single dose of 90 mg DA-9701 after fasting and had a washout period. After the washout period, 90 mg of DA-9701 was taken once at fed. The second subgroup was designed to be opposite of the first subgroup. Urine samples were collected at pre-dose (0 h) and 0–4 h, 4–8 h, 8–12 h, and 12–24 h after 90 mg DA-9701 single administration. Collected urine samples were transferred into 15 mL polypropylene tube and stored at −70 °C before analysis.

Urinary corydaline concentrations were determined using LC/MS in NexioLab (Seoul, Korea). Urinary corydaline was extracted by liquid–liquid extraction using methyl-tert-butyl-ether. UFLC-XR (Shimadzu, Kyoto, Japan), coupled with QTrap 5500 system (SCIEX, Framingham, MA, USA), was used for analysis. Sample separation was performed on Luna phenyl-hexyl column (100 × 2.00 mm, 5 μM, Phenomenex) using (a) 0.1% formic acid in distilled water and (b) acetonitrile (80:20, *v*/*v*). The calibration curve ranged from 5 to 1000 pg/mL, and three quality control samples were set to 15 (low QC), 100 (medium QC), and 750 (high QC) pg/mL, respectively. The analytical system in the validation batch showed sufficient suitability (calculated coefficient of variance: 1.99%) and linearity (correlation coefficient was >0.999). Accuracy and precision within validation batches were 97.39–106.5%.

### 2.3. Sample Preparation

Urine sample was thawed at room temperature and centrifuged at 13,000× *g* for 15 min at 4 °C. 200 μL of urine sample was transferred into a 1.5 mL centrifuge tube and four-fold volumes of deionized water were added to it. Next, the sample was vortexed gently and transferred into vials for analysis.

### 2.4. Chromatography and Mass Spectrometry Conditions

Dionex UltiMate 3000 UHPLC system coupled with orbitrap mass spectrometer (Thermo, Waltham, MA, USA) was used for metabolic profiling. Sample separation was performed on a 2.1 × 100 mm (2.6 μm) Kinetex C18 column (Phenomenex, Torrance, CA, USA). For the mobile phase, it was used deionized water containing 0.1% formic acid (A) and acetonitrile containing 0.1% formic acid (B). The column temperature was set to 40 °C, and the flow rate 0.2 mL/min. The gradient method was as follows: 5% B for 0–1 min; 30% B for 1–8 min; 70% B for 8–13 min; 95% B for 13–14 min; 95% B for 14–16 min; 5% B for 16–17 min; and 5% B for 17–20 min [16]. Pooled urine sample was used as quality control (QC) to confirm system suitability. Mass detection was carried out using Q exactive focus triple quadrupole-orbitrap mass spectrometer, with heated electrospray ionization (HESI) source in positive and negative polarity switching mode. Data were collected from 120 to 1050 *m/z* using full MS-data dependent MS^2^ (ddMS^2^) scan mode at resolution 70,000 and 17,500 for full MS and MS^2^, respectively.

### 2.5. Data Processing and Multivariate Analysis

Peak alignment and normalization by QC-based robust LOESS signal correction (QC-RLSC) method using Compound Discoverer 2.1 software (Thermo, Waltham, MA, USA). Normalized peak area was applied in the multivariate statistical analysis to identify alteration of endogenous metabolites between pre- and post-dose. A total of 90 mg of DA-9701 was administrated under the fasting statement to healthy participants and urine samples were collected at pre-dose (0 h), 0–4 h, 4–8 h, 8–12 h, and 12–24 h. Multivariate analysis was performed to search for metabolites that change at each four time points for 0 h using the SIMCA 13 software (Umetrics, Umeå, Sweden). The principal component analysis (PCA) was conducted to visualize QCs to determine how well the analysis has performed. The closely QCs clusters in the entire data were used to confirm whether there was a mechanical error or variation between different batches through the unsupervised PCA method. An orthogonal partial least squares discriminant analysis (OPLS-DA) was calculated to establish classified candidates according to the DA-9701 administration. In the OPLS-DA model, both variable importance for the projection (VIP) value ≥3.0, the absolute covariance of *p*-value ≥0.05, and *p* (correlation) value ≥0.5, which was selected for future statistical analysis. Metabolite candidates were cut-off based on MS/MS spectrum similarity of 80% or more in mzCloud, BioCyc, and ChemSpider databases included in the Compound Discoverer software. After confirming that candidates were an endogenous substance in Human Metabolome Database (HMDB), further statistical analysis was conducted using the IBM SPSS Statistics 22 package (Chicago, IL, USA).

### 2.6. Metabolites Identification

Standard materials were purchased to compare and identify the candidates. Each standard material was dissolved in the optimal solvent and analyzed at the concentration of 1 μM, similarly to the sample analysis conditions. Metabolites identification were performed under the same conditions as the studied sample. We compared the similarity between the retention time using a chromatogram and mass fragmentation pattern from standard materials and samples under study.

### 2.7. Pathway Analysis and Statistical Analysis

Pathway analysis of selected metabolites was performed through identification process. The KEGG database was used to investigate the pathway of selected metabolites. A metabolite not registered in the KEGG database was searched on HMDB to confirm its involvement in human metabolic pathways. To determine whether normalized peak area had a significant difference after the administration of extract, a statistical analysis was performed using SPSS Statistics 22 package and analyzed using the paired *t*-test and Wilcoxon signed rank test according to the results of normality test. Results were considered significant when *p* < 0.05.

## 3. Results

### 3.1. Untargeted Metabolomics Analysis and Candidate Selection

**Figure 1:**
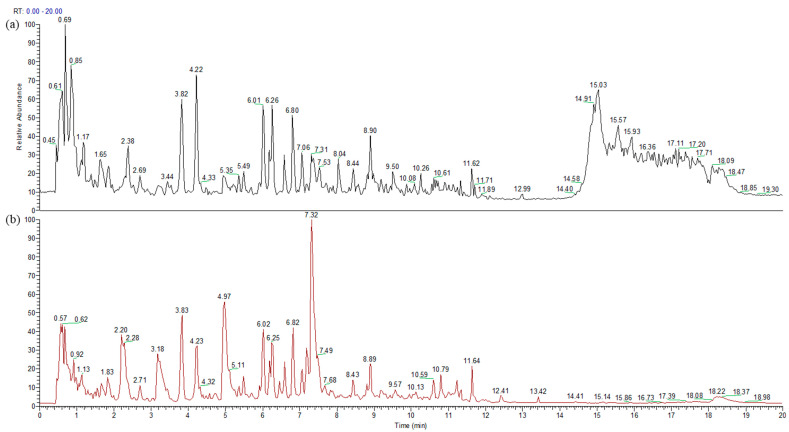
The representative chromatogram on (**a**) positive ionization mode and (**b**) negative ionization mode of one volunteer’s sample.

The representative total ion chromatogram (TIC) in both positive and negative modes is presented in Figure 1. In order to perform a multivariate analysis, raw data was processed by Compound Discoverer. As a result of the data processing, all the 6400 variables were listed from all the four groups.

**Figure 2:**
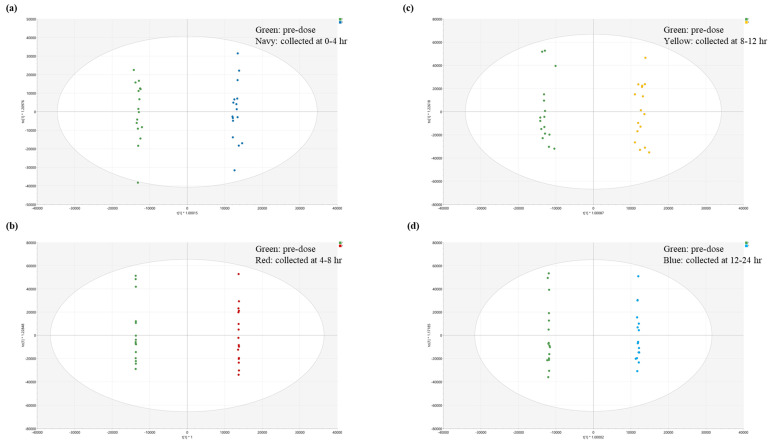
The scatter plots of orthogonal partial least squares discriminant analysis (OPLS-DA) of pre-dose and postdosing urine samples after 90 mg single dosing after fasting. Each model shows the difference between urine samples of pre-dose and (**a**) 0–4 h, (**b**) 4–8 h, (**c**) 8–12 h, and (**d**) 12–24 h after administration.

After multivariate analysis, four groups of urine sample (collected at 0–4 h, 4–8 h, 8–12 h, and 12–24 h after administration of 90 mg DA-9701) were compared with the pre-dose to select the statistically significant candidates. Total 45, 27, 19, and 15 candidates were selected for each time-point. Using Compound Discoverer, we were able to identify a spectrum similarity with 14, 11, 8, and 7 candidates in each time-point group. Next, a total of eight, nine, six, and seven candidates were searched for endogenous substances from human metabolome database (HMDB). The scatter plot of orthogonal partial least squares discriminant analysis (OPLS-DA) is presented in Figure 2.

### 3.2. Metabolite Identification

**Figure 3:**
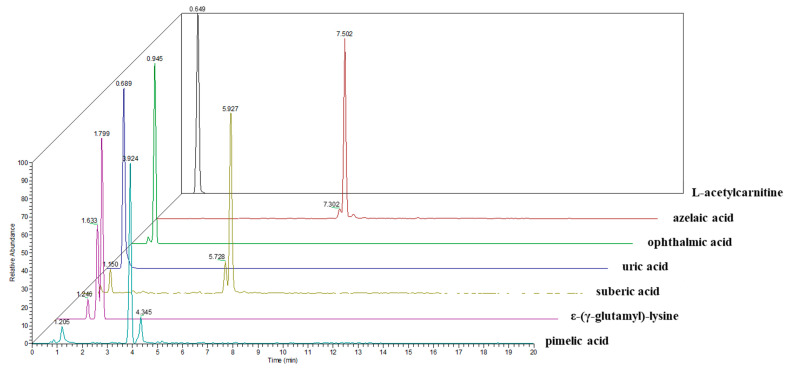
The extracted ion chromatogram (EIC) of a urine sample in full MS scan mode. Seven candidates were separated by difference colors. Black, l-acetylcarnitine; red, azelaic acid; green, ophthalmic acid; blue, uric acid; olive, suberic acid; purple, ε-(γ-glutamyl)-lysine; pale blue, pimelic acid.

The identification of metabolites was performed of the seven commercially available compounds (l-acetylcarnitine, azelaic acid, ophthalmic acid, uric acid, suberic acid, ε-(γ-glutamyl)-lysine, and pimelic acid) in the aforementioned candidates. As a result, the same fragmentation pattern was observed in each standard material and the randomly selected sample (Figure S1). The chromatogram for seven selected candidates is presented in Figure 3.

Compared spectra were matched with the reference spectrum retrieved from online databases such as mzcloud (https://www.mzcloud.org/, accessed on 10 November 2020) and HMDB (http://www.hmdb.ca/, accessed on 10 November 2020). Seven candidates were classified as metabolites and additional statistical and pathway analysis were performed (Table 2).

### 3.3. Pathway Analysis

The pathway analysis was performed using HMDB and Kyoto encyclopedia of genes and genomes (KEGG) databases for the final seven metabolites. Suberic acid, azelaic acid, pimelic acid, and l-acetylcarnitine were involved in lipid transport and metabolism, fatty acid metabolism, and lipid peroxidation. Uric acid was found to be primarily involved in purine metabolism. Based on the results of pathway analysis, most of the candidates identified in this study were considered to be involved in fundamental metabolism of various cells. The compounds ε-(γ-glutamyl)-lysine and ophthalmic acid could not find any metabolic pathways, which could relate to the other metabolites.

### 3.4. Statistical Analysis

Statistical analysis was performed to compare the QC-based normalized peak areas for seven metabolites detected in the urine with pre-dose 0–4 h, 4–8 h, 8–12 h, and 12–24 h after a single dose of 90 mg DA-9701 after fasting. Results were presented in Table 3. As a result, ε-(γ-glutamyl)-lysine and pimelic acid showed a significant difference (*p* < 0.05) at all time points. However, no other metabolites showed a significant difference at all time points. In addition, after analysis for each individual time points, there was no significant difference for the metabolites studied, except for ε-(γ-glutamyl)-lysine.

## 4. Discussion

**Figure 4:**
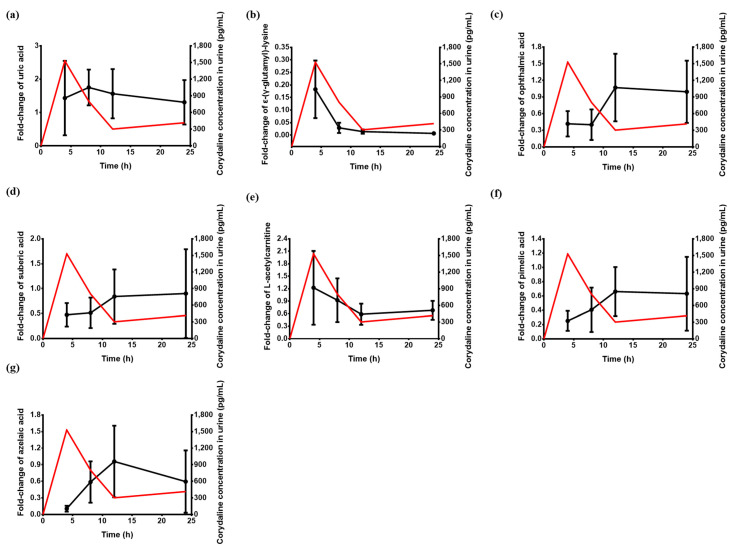
The mean concentration-time profiles of corydaline and mean fold-change of seven candidate in urine after administration of 90 mg DA-9701 (n = 16). (**a**) uric acid; (**b**) ε-(γ-glutamyl)-lysine; (**c**) ophthalmic acid; (**d**) suberic acid; (**e**) l-acetylcarnitine; (**f**) pimelic acid; and (**g**) azelaic acid. *x*-axis presented time (hours) against peak area for candidates (left *y*-axis) and corydaline concentration in urine (right *y*-axis). All data points are presented as average peak area with error bars.

In this study, we have evaluated the changes in endogenous urinary metabolites after administration of DA-9701 (Corydalis tuber and Pharbitidis seed extract), through untargeted metabolomics approaches. The raw chromatogram and mass spectra were obtained using high-resolution mass spectrometer (triple quadrupole-orbitrap mass spectrometer), with data processed using Compound Discoverer. Multivariate analysis was performed using processed peak area data. As a result, a total of 6400 variables were listed. After statistical analysis, seven candidates (l-acetylcarnitine, azelaic acid, ophthalmic acid, uric acid, suberic acid, ε-(γ-glutamyl)-lysine, and pimelic acid) were selected. Among the identified metabolites, pimelic acid and ε-(γ-glutamyl)-lysine showed significant differences at all time points after administration, when compared with pre-dose. The fold-change of uric acid was increased at all time points, and for l-acetylcarnitine, it increased during 0–4 h before it plummeted. Ophthalmic acid, pimelic acid, suberic acid, and azelaic acid accumulation decreased until 8 h and then it either increased or decreased. ε-(γ-glutamyl)-lysine accumulation levels tended to decrease continuously until 24 h after administration. The corydaline concentration-time profiles and alteration of fold-changes for candidates are shown in Figure 4.

Based on the discovered metabolites, it is considered that changes in energy metabolism following administration of DA-9701 primarily affected the improvement of gastrointestinal motility. Except for ε-(γ-glutamyl)-lysine, most of the candidates were found to affect the purine metabolic pathway, lipid, fatty acid metabolism, and lipid peroxidation. Most of the metabolite and microbiome changes in FD, previously analyzed in rats, belonged to the metabolism of energy, amino acids, nucleotides, and short chain fatty acids (SCFAs) [17]. Another study concluded that uric acid and metabolites involved in energy metabolism were somehow involved in dyspepsia, after administration of the Chinese medicine Weikangning for functional dyspepsia model rat [18].

Dicarboxylic acids (DCA), such as suberic acid, pimelic acid, and azelaic acid, increase when oxidation pathways other than β-oxidation are involved in mitochondria [19]. Compared with pre-dose, the peak intensities of the three DCAs at the C_max_ point of corydaline were significantly reduced (Figure 4). Then, we can suggest that DA-9701 administration can improve gastrointestinal motility by increasing of energy production followed by increased levels of β-oxidation.

ε-(γ-glutamyl)-lysine and pimelic acid showed significant differences at all times compared with pre-dose. ε-(γ-glutamyl)-lysine is widely distributed in fish eggs and muscle protein [20], and endogenous ε-(γ-glutamyl)-lysine is synthesized by tissue transglutaminase 2 (tTG2) or by fibrin catabolism by γ-glutamylamine cyclotransferase [21,22]. This molecule is known to be involved in several pathophysiological changes, such as fibrinogenesis and inflammatory responses [21]. However, known information on its impact on gastrointestinal motility has been very limited. Uric acid, an antioxidant, is produced when hypoxanthine and xanthine are metabolized by xanthine dehydrogenase (XDH) [23]. Inflammation caused by changes in the normal intestinal flora can induce FD; therefore, it is thought that the antioxidant effect caused by an increase in uric acid can relief FD symptoms. It was confirmed that uric acid increased by at least 1.31-fold after DA-9701 administration [24,25].

In DA-9701, compound other than corydaline and chlorogenic acids may also affect the changes of endogenous metabolites due to metabolic alteration. The methanolic extract of *Corydalis yanhusuo* tubers contained eight isoquinoline alkaloids, tetrahydropalmatine, corydaline, protopine, berberine, palmatine, jatrorrhizine, coptisine, and dehydrocorydaline [8]. Compared with tacrine, a positive control of acetylcholinesterase inhibitor (AChE inhibitor), corydaline has shown less potency to inhibit AChE. Protoberberine-type alkaloids, such as copstine, berberine, and palmatine, shown moderate inhibitory activity [8]. Natural products such as coptisine and columbamine, alkaloids extracted from *Corydalis yanhusuo,* can alleviate dyspepsia caused by bacterial infection [26,27].

The anti-inflammatory activity of berberine is also thought to be an important factor that can influence the gastrointestinal physiology and microenvironment. According to Micol Tillhon et al., berberine induces bacterial death through DNA damage and inhibits the secretion of cytokines involved in inflammatory responses, such as TNF-α (tumor necrosis factor), MCP-1 (monocyte chemo-attractant protein), and IL-6 (interleukin) [28]. In addition, the production of PGE2 (prostaglandin E2) and COX-2 (cyclooxygenase) is also reduced, thereby suppressing further the inflammatory response [28]. In several studies, the infiltration of mast cell and eosinophils or micro-inflammation have been reported to contribute to FD [29,30,31]. Relief of inflammation along the gastrointestinal tract is thought to help to improve functional dyspepsia–related symptoms. Further studies are needed to determine the correlation between the changes in endogenous metabolites following the administration of DA-9701 toward the improvement of FD symptoms. This study evaluated the pharmacokinetics of DA-9701 in healthy people. The gastrointestinal physiologies in FD patients showed to be different within healthy conditions could be due to neurological factors or immunological changes [32]. After DA-9701 administration to FD patients, gastric emptying, colonic transit, and functional constipation were improved; however, in healthy subjects, the gastric accommodation was not significantly affected [33,34]. Therefore, it was necessary to analyze the effect of the administration of DA-9701 on the pathophysiological changes in FD patients.

## 5. Conclusions

In conclusion, a metabolic profiling was performed using HRMS for urine samples obtained after administration of DA-9701. As a result, >6000 candidates were analyzed and a final group of seven endogenous urinary metabolites were selected. The pathway analysis of the seven metabolites was mostly associated with lipid metabolism. Further studies on a potential association between prokinetic activity and the lipid metabolic pathway are needed. A biomarker that can predict a drug’s response may help to establish a more effective pharmacotherapeutic regimen or figure out the action of mechanisms.

## Figures and Tables

**Table 1:** Summary of characteristics of each study group.

Characteristics	Group A	Group B	Group C
	(*n* = 4)	(*n* = 6)	(*n* = 6)
Age	24.25 ± 1.50	23.83 ± 1.47	25.33 ± 3.78
	(23–26)	(22–26)	(20–30)
Height (cm)	174.9 ± 5.08	171.9 ± 2.26	173.6 ± 7.37
	(168.0–179.1)	(169.3–175.4)	(162.2–183.5)
Weight (kg)	68.35 ± 10.5	67.65 ± 8.90	72.32 ± 14.4
	(59.3–77.6)	(56.4–83.8)	(58.3–98.4)
BMI (kg/m^2^)	22.23 ± 2.37	22.83 ± 2.74	23.78 ± 2.89
	(19.5–24.4)	(19.6–27.9)	(21.0–29.2)

All the data were represented mean ± standard deviation (range). Group A: Administered 90 mg DA-9701. Group B: (phase I) 90 mg DA-9701 administered before meals/washout period/ (phase II) 90 mg DA-9701 administered after meals. Group C: (phase I) 90 mg DA-9701 administered after meals/washout period/ (phase II) 90 mg DA-9701 administered before meals.

**Table 2:** The candidates list in the urine samples after single dosing 90 mg DA-9701 after fasting.

Metabolites	Retention Time (min)	Molecular Weight (g/mol)	VIP Value	Related Pathway
l-acetylcarnitine	0.66	204.12303	5.66	lipid transport and metabolism
				fatty acid metabolism
				lipid peroxidation
Azelaic acid	7.44	188.10429	4.75	lipid transport and metabolism
				fatty acid metabolism
				lipid peroxidation
Ophthalmic acid	0.94	289.12739	3.92	unclear
Uric acid	0.68	168.02844	3.69	purine metabolism
Suberic acid	5.95	174.08862	3.69	lipid transport and metabolism
				fatty acid metabolism
				lipid peroxidation
ε-(γ-glutamyl)-lysine	1.83	275.14819	3.26	unclear
Pimelic acid	4.09	160.07276	3.17	lipid transport and metabolism
				fatty acid metabolism
				lipid peroxidation

VIP, variable importance for the projection; VIP values were obtained from the OPLS-DA model.

**Table 3:** The mean fold-changes for eight metabolites in the urine sample after 90 mg DA-9701 single administration at fasting state.

Identified	0–4 h	4–8 h	8–12 h	12–24 h
Candidates				
Uric acid	1.43 ± 1.12	1.75 ± 0.53 *	1.56 ± 0.74 *	1.31 ± 0.67
ε-(γ-glutamyl)-lysine ^#^	0.18 ± 0.11 *	0.03 ± 0.02 *	0.01 ± 0.01 *	0.01 ± 0.01 *
Ophthalmic acid	0.42 ± 0.23 *	0.40 ± 0.28 *	1.07 ± 0.61	0.99 ± 0.56
Pimelic acid ^#^	0.25 ± 0.14 *	0.41 ± 0.31 *	0.66 ± 0.34 *	0.63 ± 0.52 *
Suberic acid	0.48 ± 0.23 *	0.51 ± 0.30 *	0.84 ± 0.54	0.90 ± 0.89
Azelaic acid	0.11 ± 0.05 *	0.59 ± 0.37 *	0.96 ± 0.65	0.60 ± 0.56 *
L-acetylcarnitine	1.22 ± 0.89	0.92 ± 0.52	0.59 ± 0.25 *	0.68 ± 0.23 *

All the data were represented mean ± standard deviation and compared with pre-dose. * significant difference compared with pre-dose (*p* < 0.05); ^#^ significant differences at all time points.

## Data Availability

All data are available upon request.

## Supplementary Materials

The following are available online at https://www.mdpi.com/article/10.3390/pharmaceutics13040522/s1. Figure S1. Mass spectra of the selected seven candidates and comparison with standard compounds: (a) l-acetylcarnitine, (b) azelaic acid, (c) ophthalmic acid, (d) uric acid, (e) suberic acid, (f) ε-(γ-glutamyl)-lysine, and (g) pimelic acid.
