# Supplementary material for: Urinary Metabolomic Profiling after Administration of Corydalis Tuber and Pharbitis Seed Extract in Healthy Korean Volunteers
Source: Pharmaceutics. 2021 Apr 9;13(4):522. doi: 10.3390/pharmaceutics13040522 (PMC8069993; doi:10.3390/pharmaceutics13040522)
Supplement: Supplementary file 1 [file pharmaceutics-13-00522-s001.pdf]

pharmaceutics

MDPI

Supplementary Materials: Urinary Metabolomic Profiling after Administration of Corydalis Tuber and Pharbitis Seed Extract in Healthy Korean Volunteers

Hyeon-Cheol Jeong, Jung Eun Park, Yohan Seo, Min-Gul Kim * and Kwang-Hee Shin *

(a)

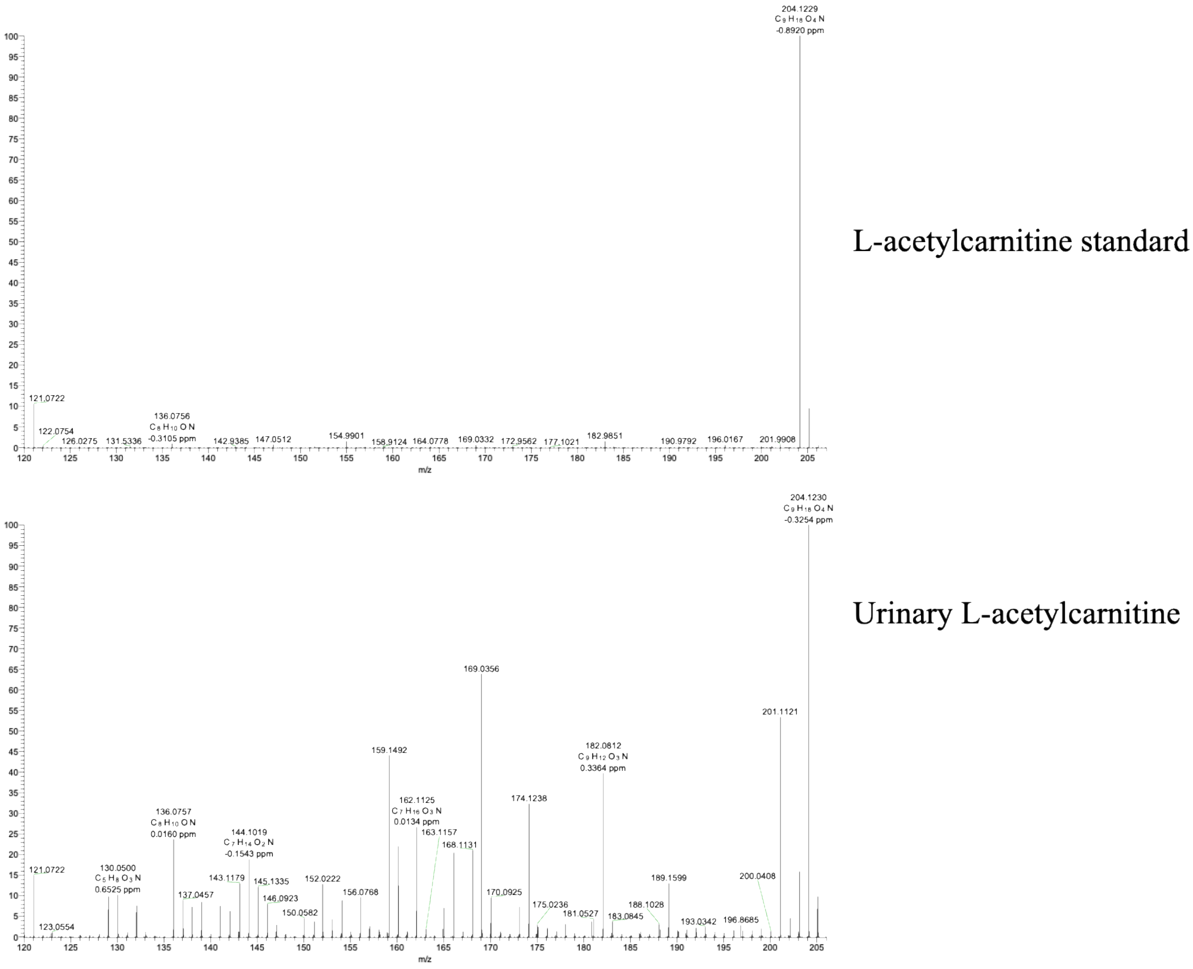

(b)

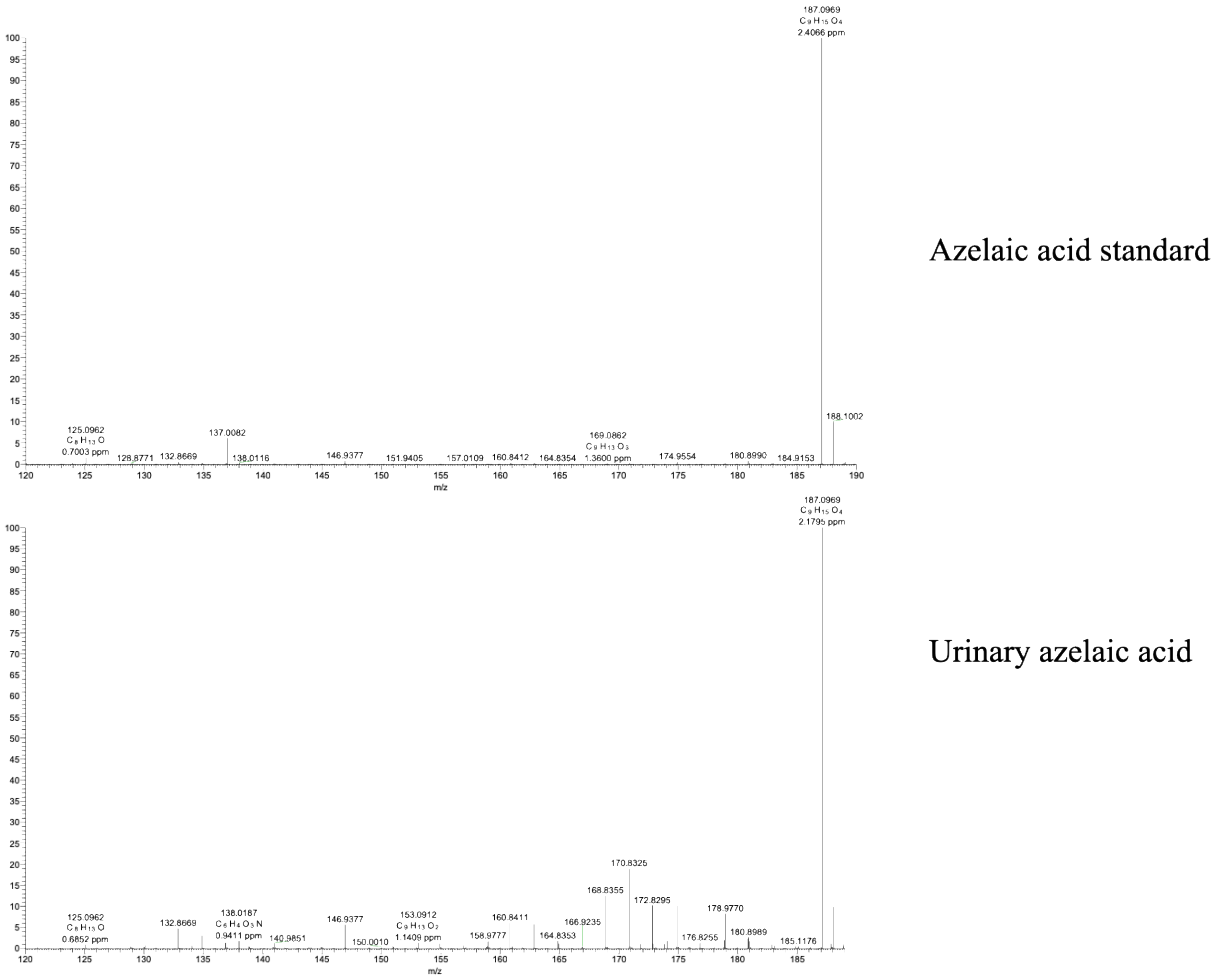
187.0969
$C_9H_{15}O_4$
2.4066 ppm
Azelaic acid standard
m/z
187.0969
$C_9H_{15}O_4$
2.1795 ppm
Urinary azelaic acid
m/z

(c)

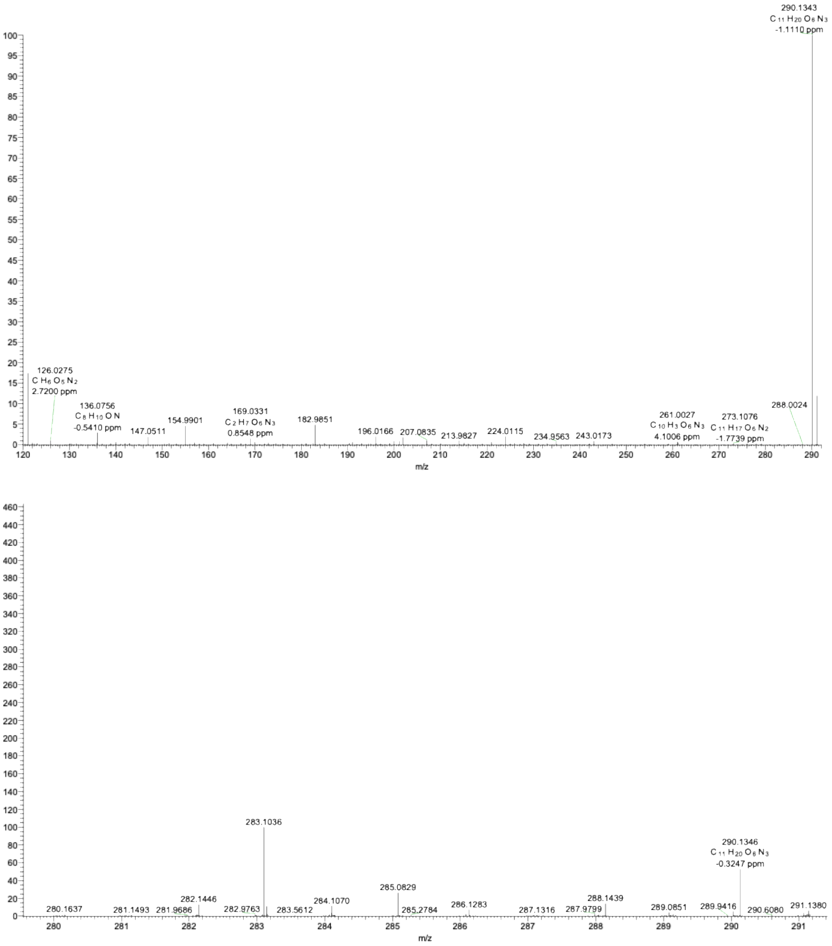

Ophthalmic acid standard

Urinary ophthalmic acid

(d)

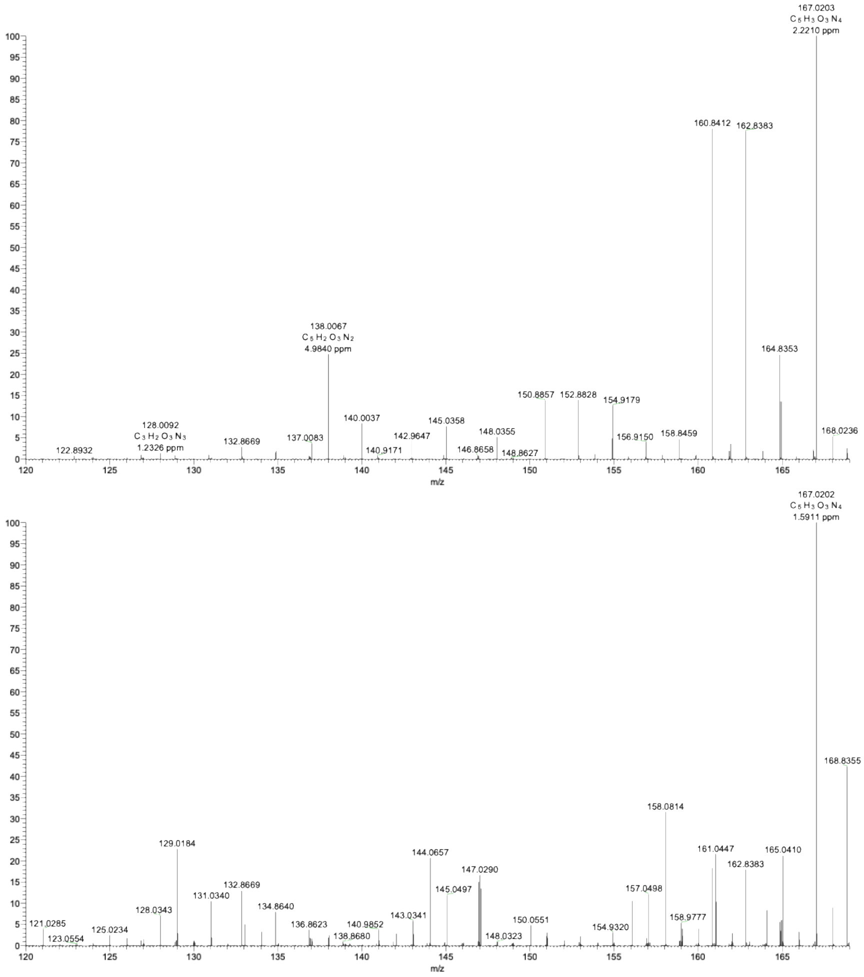

Uric acid standard

Urinary uric acid

(e)

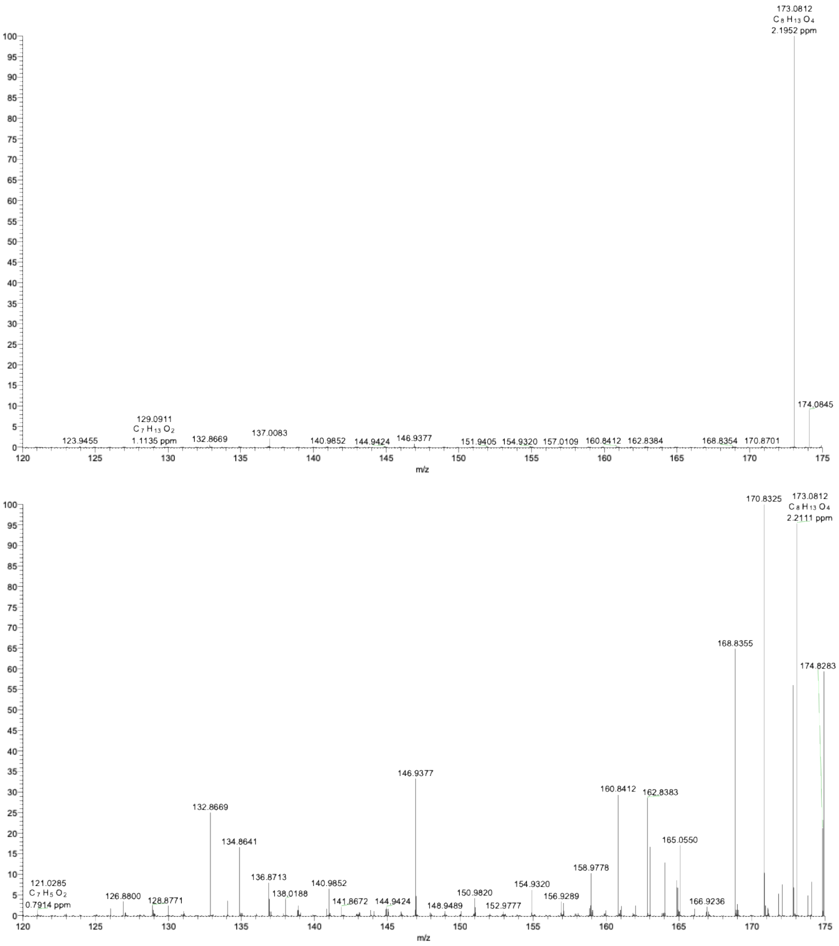

Suberic acid standard

Urinary suberic acid

(f)

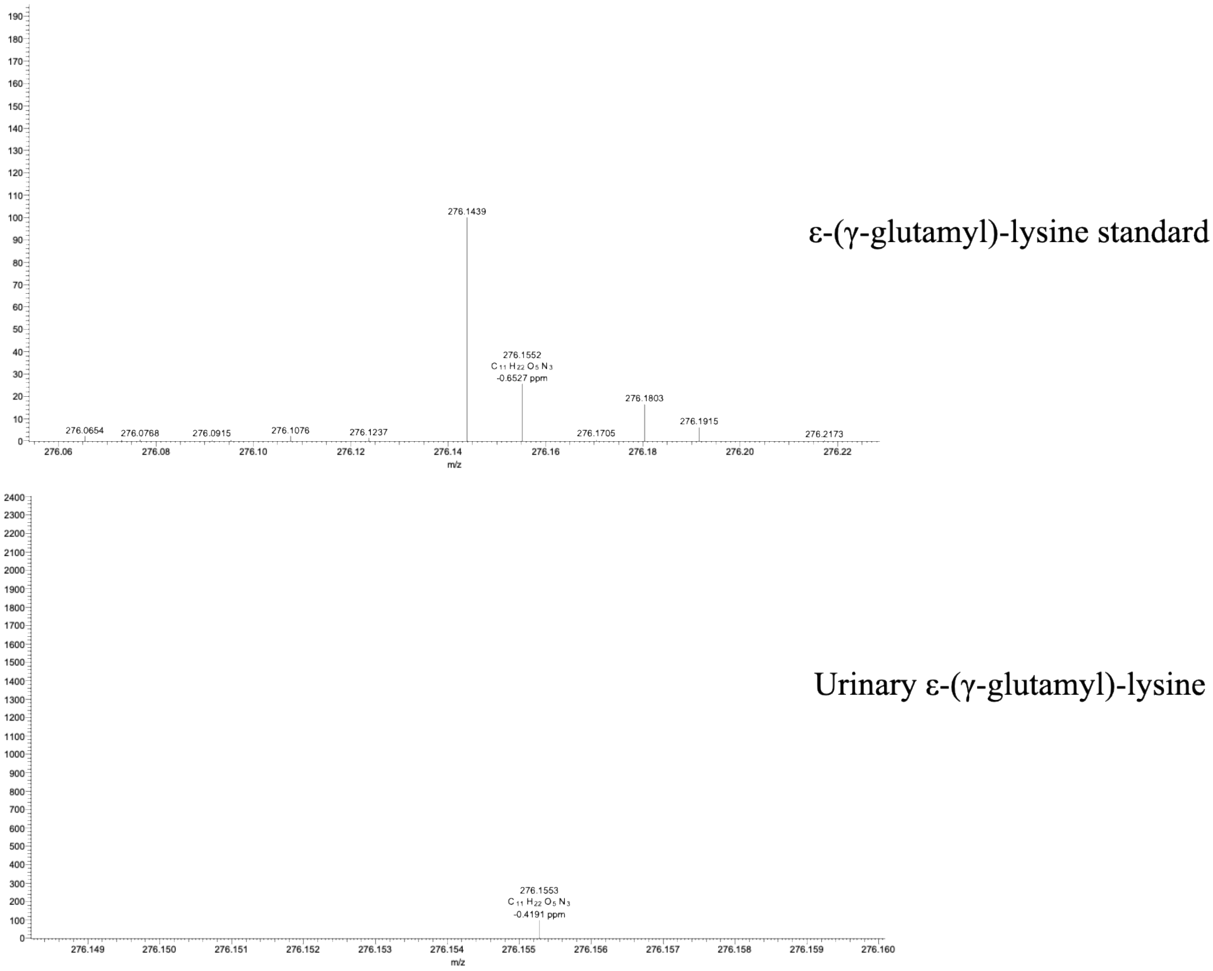
276.1439
276.1552
C 11 H 22 O 5 N 3
-0.6527 ppm
276.1803
276.1915
276.0654
276.0768
276.0915
276.1076
276.1237
276.1705
276.2173
m/z
ε-(γ-glutamyl)-lysine standard
276.1553
C 11 H 22 O 5 N 3
-0.4191 ppm
m/z
Urinary ε-(γ-glutamyl)-lysine

(g)

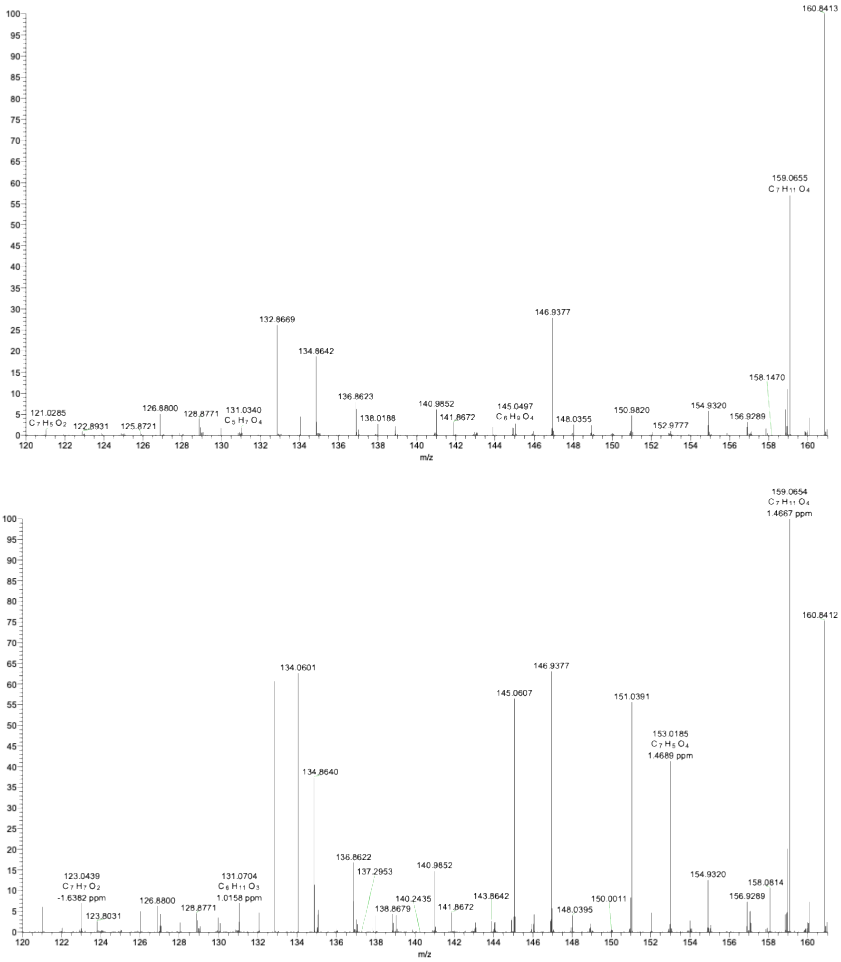

Pimelic acid standard

Urinary pimelic acid

Figure S1. Mass spectra of the selected seven candidates and comparison with standard compounds: (**a**) L-acetylcarnitine, (**b**) azelaic acid, (**c**) ophthalmic acid, (**d**) uric acid, (**e**) suberic acid, (**f**) ε-(γ-glutamyl)-lysine, and (**g**) pimelic acid.
